# Preschool teachers display a flexible pattern of pedagogical actions in promoting healthy habits in children

**DOI:** 10.3389/fpsyg.2023.1172460

**Published:** 2023-04-24

**Authors:** Verónica Violant-Holz, Carlota Rodríguez-Silva, Manuel J. Rodríguez

**Affiliations:** ^1^Department of Didactics and Educative Organization, Faculty of Education, Universitat de Barcelona, Barcelona, Spain; ^2^International Observatory in Hospital Pedagogy, Universitat de Barcelona, Barcelona, Spain; ^3^Research Group and Innovation in Designs (GRID), Technology and Multimedia and Digital Application to Observational Designs, Thematic Core, Hospital Pedagogy, Barcelona, Spain; ^4^Department of Biomedical Sciences, Institute of Neurosciences, School of Medicine and Health Sciences, Universitat de Barcelona, Barcelona, Spain; ^5^Institut d’Investigacions Biomèdiques August Pi i Sunyer (IDIBAPS), Barcelona, Spain

**Keywords:** preschool teachers, teaching strategies, health education, healthy school environment, ESAR model, CHAID analysis

## Abstract

The school represents the optimal setting for promoting the physical, emotional, and social health of children, especially during the first years of life. Understanding the pedagogical actions of teachers to address health education is an important first step in promoting healthy behaviors in children. We inhere analyzed the pedagogical action patterns in the preschool teaching of healthy habits from a holistic health perspective. We used photography as a strategy for data collection and applied a Chi-square automatic interaction detection (CHAID) classification tree, a data mining procedure, to generate a pattern model. We found that the school space and the learning playfulness strategies for the development of executive functions, classified according to the exercise, symbolic, assembly, rules (ESAR) model, were the main factors that influence the pedagogical actions fostering healthy habits. By contrast, the school and the pedagogical resources of the classroom are factors with a much smaller impact on working with healthy habits. This pedagogical action pattern is flexible, since teachers conduct a multiplicity of pedagogical actions through different strategies, in different school spaces, at any time. In conclusion, our results unmask the interdependent relationships between the different factors that determine the teacher’s actions at the preschool. It also contributes to the understanding of the teacher’s practices in fostering healthy habits in a healthy learning environment.

## 1. Introduction

The sustainable development goals of the United Nations proposed education as a crucial factor for improving individual quality of life (World Health Organization, 2003). UNESCO is implementing a global strategy on Education for Health and Well-being (UNESCO, 2022). Early childhood represents a critical period for intervention because healthy lifestyle habits are developed during this period (Goldfield et al., 2012). The school represents the optimal setting to promote physical, emotional, and social health, especially during the first years of life (Violant-Holz et al., 2021). Consequently, teachers and all the stakeholders involved in education have been requested to systematically consider how educational approaches could increase the healthy behaviors of children (Pulimeno et al., 2020), understanding them as daily healthy routines.

As a consequence, some studies encourage preschool teachers to foster healthy habits within children, such as promoting mental health (Haraldsson et al., 2017; Blewitt et al., 2020), invigorating healthier nutrition and feeding (Whiteside-Mansell and Swindle, 2019), animating physical activity (Tyler et al., 2020), inciting hygiene (Bonnesen et al., 2015), or reassuring emotional health (Ocasio et al., 2015). Recent research encourages preschool teachers to integrate all those health dimensions in their educational approach in classrooms (Violant-Holz et al., 2021), following the emerging concept of health education (Gavidia, 2016). This means understanding education, and therefore also health education, as a complex phenomenon (Morin et al., 2002), integrating physical, mental, and social health with the frailty concept (Boers and Cruz Jentoft, 2015). However, to our knowledge, there are no studies using data mining to analyze the characteristics of the pedagogical actions and the patterns of interaction with children deployed by teachers to address these education-for-health approaches.

Photography is a classical visual method in social science research, including the education field (Deligny, 2006). For example, photography can be used as a method reflecting healthy habits implemented in real-time, in terms of educational spaces in the whole school (Lewallen et al., 2015). Currently, visual methods can be understood as tools for both eliciting data and as a source of data. In the first sense, we can enumerate research using visual methods for eliciting data for educative purposes. This includes cartographies (Padilla-Petry et al., 2021); photo-elicitation (Dworkin et al., 2021), which was also successfully used regarding health concepts with children and adolescents with disabilities, such as health and well-being, physical activity participation and leisure activity (Lumia et al., 2021); photo-interview (Kolb, 2008); or photo-voice (Shaw, 2021), which may also constitute an inclusive didactic strategy (Parrilla et al., 2017).

In the second sense, photographs are used as an empirical tool for recording information in educational research wherever the child is. Thus, photo evidence has effectively been used as a tool to approach healthy eating within families through remote food photography (Bekelman et al., 2019; McCloskey et al., 2019) and proven a feasible methodology to capture parent-packed lunches for preschoolers (Elliott et al., 2021). Other studies applied photo evidence to assess the eating behaviors of scholars. It has been used as a source of data to analyze healthy eating within schools (Bélanger et al., 2016), to compare the nutritional quality of morning snacks (Bucher Della Torre et al., 2021), to measure what students at elementary schools select and consume from school cafeteria meals (Swanson, 2008), and to evaluate what items children chose from the salad bar at schools (Moreno-Black and Stockard, 2018). Photography has also been applied to examine the characteristics of effective drinking water access in schools (Patel et al., 2020).

Beyond nutrition, Kontak et al. (2017) collected photographic data to examine school-based health environments in an enlightening study. They collected photographs to analyze different health dimensions, which include promotion, access, availability of opportunities for healthy eating and physical activity, healthy school climate and safety, and school accessibility. All in all, photography allows us to see what was happening at a particular point in time (Cleland and MacLeod, 2021), and it has been proven an effective method to capture the reality of the schools in education research, for example by the perspectives of children (Kyrönlampi et al., 2021).

Data mining is an analysis method that extracts information from large data sets, enables the construction of predictive and classification models, and detects interactions within subsets of variables (Collins, 2021). In the education field, several data mining strategies such as classification trees, neural networks, and logistic regression have been implemented to generate classification models of students’ profiles and teachers’ performance (See Khan and Ghosh, 2021 for a review). The decision tree for classification and prediction is considered as an effective quantitative method for the analysis of condensed and categorized qualitative data (Collins, 2021). The predictive ability of this classification method makes it a popular choice for student performance analysis (Qasrawi et al., 2020; Khan and Ghosh, 2021), and educational quality performance assessment (Cervero, 2020). For example, decision trees have provided sufficient accuracy in predicting education tasks for sports learning (Feu et al., 2019). Information unmasked by education data mining procedures helps researchers to identify new patterns, which are valuable in evaluating pedagogical actions (Rodrigues et al., 2018).

Decision tree algorithms have been used for both the classification and prediction of factors in relationship with children’s health. For example, they have been used for the analysis of the underlying causes of child hunger among households accessing food pantries (Wetherill et al., 2021), for the prediction of risk factors for obesity among children aged 24 to 80 months (Lee et al., 2019), and for the identification of success indicators in the implementation in public schools of a physical activity program (Nickelson et al., 2011). Some other studies used decision tree analysis to identify predictors that allow us to implement measures related to psychological health in schools, such as the prevention of homophobic victimization (Fisher and Tanner-Smith, 2016), and bullying (Brewer et al., 2018). This analysis has also been used to detect children’s unsafety in families with parental or child developmental problems (van der Put et al., 2016). However, to our knowledge, no studies used data mining analysis to classify the healthy habits-related pedagogical actions and the patterns of interaction with children deployed by preschool teachers.

Our objective and main goal in the present study was to quantitatively define the pattern and trends of the pedagogical actions (understanding patterns as a way of interacting with children in the school) deployed by preschool teachers to promote healthy habits among children. To do that, we performed a novel mixed-method analysis that used photography as a strategy to collect qualitative data and analyzed the patterns of pedagogical actions by using a quantitative classification tree algorithm based on Chi-square automatic interaction detection (CHAID) analysis.

## 2. Methods

### 2.1. Design of the study

This study is part of a larger research assessing the impact of a co-created educational project to analyze the health-related preschool teacher’s actions and classroom routines and to improve children’s healthy habits (Violant-Holz et al., 2021). In the present study, we used a mixed-methods research design that combined observational methodology (Anguera et al., 2020), and selective methodology (Anguera and Violant, 2015) to collect qualitative data, analyze it, and integrate it into quantitative data (Portell et al., 2015; Anguera et al., 2021; Collins, 2021). We implemented the photography method as a strategy of data collection based on overt indirect nonparticipating naturalist systematic observation, guided by a deductive checklist (Anguera-Argilaga, 1979; Anguera et al., 2020). According to these authors, “Overt” means that we observed the school daily life, being openly and visibly observing; “Indirect” means that we collected photographs; “Nonparticipating” refers to minimizing interaction with participants; “Naturalistic” means that we conducted the observations in the natural environment; and “Systematic” means that researchers entered the field with an observation instrument and that this instrument guided them to perform the observations and take pictures. In this study, photographs function as empirical data collected by the researcher (Poveda, 2018). After qualitative data condensation and categorization, health-related pedagogical actions and routines at preschool were analyzed by data mining procedures.

### 2.2. Participants

Participants were recruited from four public schools in Barcelona (Spain). For enrollment of teachers, schools were approached in collaboration with the Consorci d’Educació de Barcelona, which is the main educational authority for the city’s organization of primary education centers. These four public schools were selected by non-probabilistic convenience sampling, according to their availability and willingness to participate in the study, and their allocation in different city neighborhoods, thus seeking the inclusion of participants from different socioeconomic backgrounds of the city. Teachers were selected by non-probabilistic convenience sampling from those four schools voluntarily. From a total of 21 invited teachers, 16 teachers (12 female and 4 male) and 328 children (3–6-year-old) ultimately participated throughout the entire study.

### 2.3. Instruments and procedure

For qualitative analysis of the teachers’ actions, observations were performed to capture photographs in different school spaces (Anguera et al., 2018), with a deductive checklist (Supplementary Table 1) that implied partial perceptibility. Seven trained observers took the photographs. Two of these observers trained the others in the checklist use. During the actual data collection, a coordinator provided feedback on data quality and assessment criteria (Fernández-Ballesteros, 1992). To analyze the health-related pedagogical actions of teachers, data collection was performed during different periods of the scholar course: in June and October. Observers followed the deductive checklist in the observation and taking photographs (deductive procedure). They also captured actions related to healthy habits that emerged from being at the schools (inductive procedure). These moments were analyzed following the checklist. Teachers and children knew in advance the dates and times when observers would be present to collect data. To minimize their interactions with teachers or children, observers stood at the back of the classroom during the data collection process. The first session was conducted at each school in June 2019, before the teachers’ attendance at our health-promotion project, while the second session was conducted in October 2019, after the teachers attended the project. Photographs were taken from 09.00 am until 1.30 pm.

Four hundred and fifty-two photographs were taken and reviewed to assess if they matched the criteria to be included in the analysis. These inclusion criteria were (1) to be taken in the school spaces: classrooms, corridors, playgrounds, and bathrooms; (2) to be related to the health dimensions: nutrition, physical activity, hygiene, and emotional health, and (3) to be related with pedagogical actions directly or indirectly promoted by teachers. By contrast, the criteria to exclude photographs from the study were: (1) to be repeated photographs, considered such as those photographs that did not provide new information; (2) to be a blurred picture and the actions photographed could not be distinguished in the image, and (3) to show a pedagogical action promoted by non-teacher staff, such as canteen monitors. A final number of two hundred and twenty-two photographs were included in the analysis. Two hundred and twenty photographs were excluded, because either they were repeated, had the same information class, with the same children and class, or were blurred.

### 2.4. Data classification

Each photograph was categorized into 6 nominal variables: (1) Health Dimension, (2) Educational Resource, (3) ESAR Facets, (4) Spaces, (5) Month, and (6) School, using consensus agreement between two researchers (Arana et al., 2016). As photographs are a multidimensional instrument (Cristancho and Helmich, 2019), can give information about different variables, according to the items analyzed. We controlled the mutual exclusivity criterion (Arnau et al., 1990). These categories included in each nominal variable are explained below.

#### 2.4.1. Health dimension

This nominal variable referred to the health dimensions defined by parents, teachers, and health professionals in three independent co-creation sessions (Violant-Holz et al., 2021). These dimensions defined four categories: (1) *Physical Activity*, (2) *Hygiene*, (3) *Nutrition*, and (4) *Emotional Health*. In concordance with the objectives of the current study, we selected this variable as the dependent one.

#### 2.4.2. Educational resource

This variable referred to the resources that teachers mobilized during their pedagogical actions. Following the criteria published by Torre et al. (2004) on the types of educational resources, this nominal variable had eight categories: (1) *Body*, involvement of any part of the body as a resource; (2) *Experimental materials*, such as experimentation with real, symbolic objects, technological objects and pieces; (3) *Food*, involvement of any food; (4) *Free play*, involvement of non-directed activity, wherever the child is; (5) *Informational materials*, with elements that become vehicles for learning, such as the use of photographs, a book or an image; (6) *Natural environment*, with elements found in nature and environment, such as water; (7) *Social environment*, with elements involving interpersonal relationships, such as conducting an assembly, group work, and lining up, and (8) *Worksheets*, actions involving papers and pencils.

#### 2.4.3. ESAR facets

This variable referred to the learning playfulness strategies of the Exercise, Symbolic, Assembly, Rules (ESAR) model. This model is based on childhood learning from the perspective of the child’s psychological development, which teachers addressed with their pedagogical action (Garón et al., 1996). We selected the ESAR model because it is based on pedagogical principles regarding the use of games and playfulness as learning strategies for the development of both cognitive function and personality at early ages (Piaget, 1989). Every one of the six facets of the ESAR model was considered as a category of this nominal variable: (1) *Affective behavior*, the action involved affective-emotional development in its different levels: confidence, autonomy, initiative, etc. (2) *Cognitive behavior*, the action was represented by cognitive development and sensory-motor and symbolic-intuitive behaviors; (3) *Functional ability*, the action was represented by the expression of cognitive behaviors and basic tools for living in their different forms: discovery, reproduction, efficiency, and combination; (4) *Language ability*, the action was represented by receptive and productive language, oral and written; (5) *Ludic activity*, the action was represented by play and playful behavior; and (6) *Social activities*, the action was represented in social or group context.

#### 2.4.4. Spaces

We defined this variable to assess the putative influence of the school rooms on the teacher’s pedagogical actions. This nominal variable included five categories: (1) Classrooms; (2) Corridors; (3) Playgrounds; (4) Bathrooms; and (5) Psychomotricity rooms.

#### 2.4.5. Month

This variable referred to the month when the photographs were taken. It provided information on the effect of the health promotion project on the teacher’s pedagogical actions. This nominal variable included two categories. (1) *June*, for photographs taken in June 2019, before the teachers participated in the project, and (2) *October*, for photographs taken in October 2019, after the teacher’s participation in the project.

#### 2.4.6. School

This variable referred to the school where the picture was taken. It provided information on the putative differences in pedagogical actions between the centers where the data was collected. As photographs were collected in four different schools, this nominal variable had four categories: (1) School 1; (2) School 2; (3) School 3; and (4) School 4.

**Figure 1:**
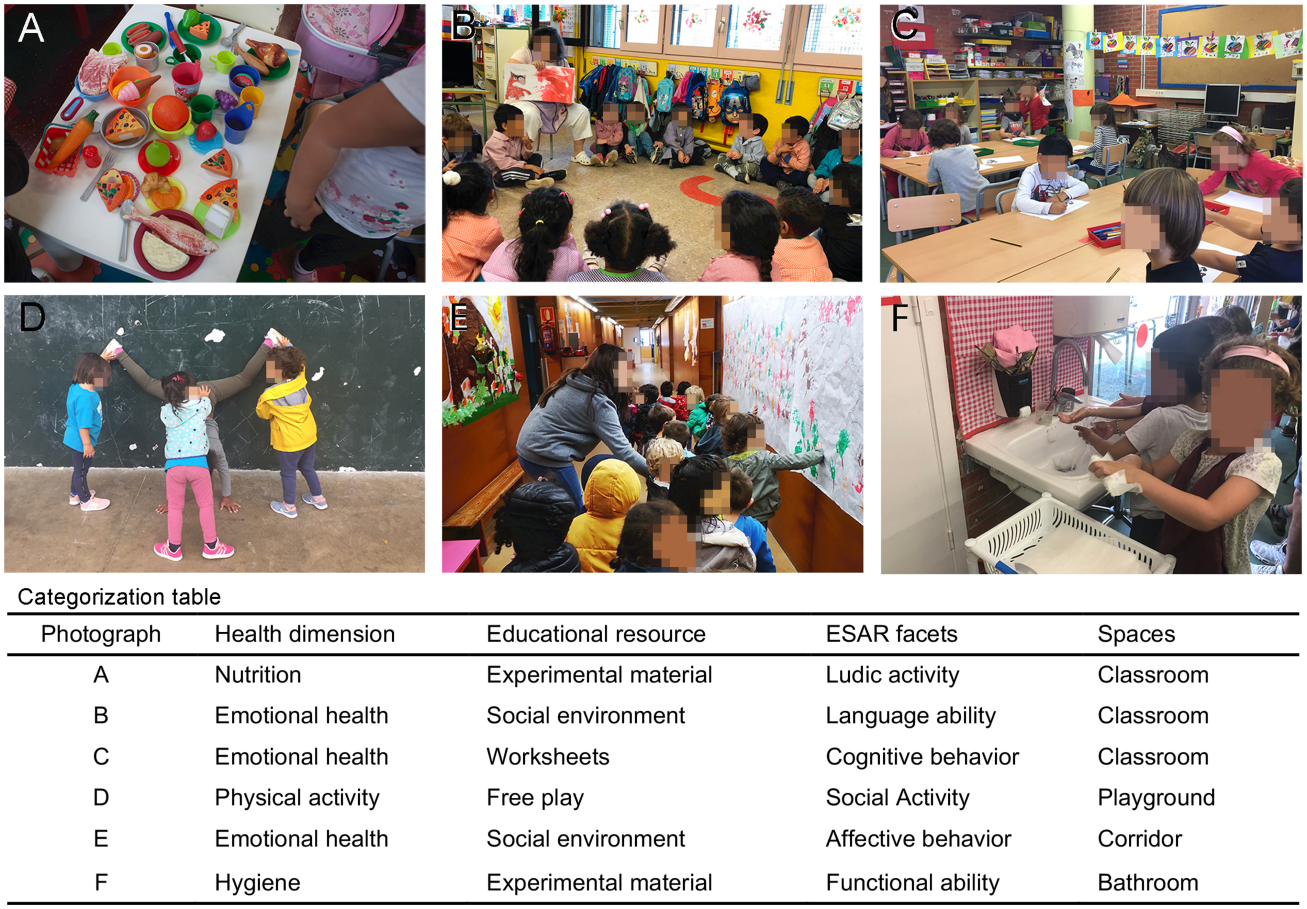
Classification of photographs into the different categories of four variables of the study. **(A–F)** Illustrative photographs taken in the study; Written informed consent to take photographs at the school was obtained from the individuals participating in this study and /or their representatives; people’s faces have been blurred to ensure privacy. The categorization table shows the classification of these six photographs into the categories of the health dimension, educational resource, ESAR Facets, and Spaces variables. See text for details.

By way of clarification, Figure 1 shows the classification of illustrative photographs into the different categories of the Health Dimension, Educational Resource, ESAR Facets, and Spaces variables.

### 2.5. Data analysis

The variables presenting the strongest impact on the pedagogical actions addressed to foster the children’s healthy habits were determined using the data mining method of the CHAID decision tree, based on the data extracted from photographs. The categorical dependent variable was Health Dimension. Firstly, we performed a descriptive exploratory analysis of every variable that defined the pedagogical actions according to the Health Dimensions. We present the number and percentage of items in each variable category in contingency tables. We then performed an inferential analysis to identify the relationships among the study variables. We performed Pearson’s Chi-squared test (*χ*^2^) to analyze the independence between the categorical variables. We assessed the association strength by calculating the Cramer’s V coefficient (*φ*c). In all analyses, we provide the exact value of *p* and considered value of *p* < 0.05 significant.

Finally, we generated a classification tree as a predictive model to classify and segment the relationships among variables (Collins, 2021). We used the CHAID algorithm to generate the classification tree because most variables were nominal and not binary (Kass, 1980). We used the exhaustive CHAID method, which allows us to analyze with precision all possible results, independently of the categories of the variables (Biggs et al., 1991). We applied the following criteria for the exhaustive CHAID tree growing: statistical test for determining node splitting, Pearson’s *χ*^2^; significance level *p* < 0.05 with Bonferroni adjustment; maximum tree depth of 5 levels; a minimum of 80 cases in the parental node; a minimum of 40 cases in the filial node; and cross-validation. All analyses were performed with the SPSS Statistics v26 (IBM Corp. USA) statistical package.

## 3. Results

### 3.1. Descriptive analysis of the model

The final number of 232 selected photographs provided a total of 267 unities of analyzed data reflecting the pedagogic actions on healthy habits at schools. Comparisons of data revealed differences in frequency proportions between health dimensions (*χ*^2^ = 52.44; d.f. = 3; *p* = 0.00001). Emotional Health presented a higher proportion of items collected (42.32%) than expected for uniformly distributed proportions (25% each), while Hygiene and Physical Activity presented lower proportions than expected (16.10 and 14.61%, respectively). The proportion of items from Nutrition was as expected (26.97%).

We then established the strength of the relationship between the variables Educational Resource, ESAR Facets, Spaces, Month, and School with the Health Dimension variable. With this analysis, we established a rank list of the variables according to their relevance for further analysis in the classification tree. We, thus, examined differences in the Health Dimension distribution among groups of Educational Resources, ESAR Facets, Spaces, Month, and School by the Pearson *χ*^2^ test (Table 1). We found differences in Health Dimension frequency distributions among all the five variables analyzed. The variable with the most statistically significant effect on the Health Dimension frequency distribution was Spaces (*p* = 1.7 × 10^−25^), with a moderate degree of association between the variable categories (*φ*c = 0.314). Educational Resources also presented a highly significant effect (*p* = 2.8 × 10^−22^), with a moderate degree of association between categories (*φ*c = 0.439), and the same was true for ESAR Facets (*p* = 5.15 × 10^−16^; *φ*c = 0.366). We observed a lower significant effect of the variables Month (*p* = 0.001) and School (*p* = 0.020) in the Health Dimension distribution frequencies, both with a low degree of association between categories (*φ*c = 0.262 for Month, and *φ*c = 0.155 for School). Nevertheless, as Health Dimension presented a significant dependence relationship with all five variables (all *p*-values were lower than 0.05), we included all of them as predictors in the further classification tree analysis.

**Table 1:** Cross-tabulation of the study independent variables with the health dimensions.

		Health dimension (%)				Total				
Variable	Domain	Physical activity	Hygiene	Nutrition	Emotional health	*n*	%	*χ* ^2^	*p*-value	*φ*_c_
Educational resources								154.15	2.8 × 10^−22^	0.439
	Worksheets	0 (0.0)	0 (0.0)	2 (2.8)	15 (13.3)	17	6.4			
	Body	5 (12.8)	8 (18.6)	0 (0.0)	12 (10.6)	25	9.4			
	Experimental material	1 (2.6)	19 (44.2)	20 (27.8)	20 (17.7)	60	22.5			
	Food	0 (0.0)	0 (0.0)	14 (19.4)	4 (3.5)	18	6.7			
	Free play	22 (56.4)	0 (0.0)	2 (2.8)	16 (14.2)	40	15.0			
	Informational material	11 (28.2)	10 (23.3)	29 (40.3)	28 (24.8)	78	29.2			
	Natural environment	0 (0.0)	6 (14.0)	4 (5.6)	5 (4.4)	15	5.6			
	Social environment	0 (0.0)	0 (0.0)	1 (1.4)	13 (11.5)	14	5.2			
	Total	39 (100)	43 (100)	72 (100)	113 (100)	267	100			
ESAR facets								107.36	5.15 × 10^−16^	0.366
	Affective behavior	1 (2.6)	4 (9.3)	1 (1.4)	28 (24.8)	34	12.7			
	Cognitive behavior	5 (12.8)	3 (7.0)	9 (12.5)	17 (15.0)	34	12.7			
	Functional ability	3 (7.7)	17 (62.8)	18 (25.0)	13 (11.5)	61	22.8			
	Language ability	8 (20.5)	6 (14.0)	24 (33.3)	27 (23.9)	65	24.3			
	Ludic activity	19 (48.7)	2 (4.7)	19 (26.4)	13 (11.5)	53	19.9			
	Social activity	3 (7.7)	1 (1.4)	1 (1.4)	15 (13.3)	20	7.5			
	Total	39 (100)	43 (100)	72 (100)	113 (100)	267	100			
Month								18.33	0.001	0.262
	June	11 (28.2)	19 (44.2)	18 (25.0)	61 (54.0)	109	40.8			
	October	28 (71.8)	24 (55.8)	54 (75.0)	52 (46.0)	158	59.2			
	Total	39 (100)	43 (100)	72 (100)	113 (100)	267	100%			
School								19.34	0.020	0.155
	School 1	5 (12.8)	10 (23.3)	24 (33.3)	18 (15.9)	57	21.3			
	School 2	10 (25.6)	6 (14.0)	13 (18.1)	28 (24.8)	57	21.3			
	School 3	4 (10.3)	10 (23.3)	13 (18.1)	32 (28.3)	59	22.1			
	School 4	20 (51.3)	17 (39.5)	22 (30.6)	35 (31.0)	94	35.2			
	Total	39 (100)	43 (100)	72 (100)	113 (100)	267	100			
Space								147.62	1.7 × 10^−25^	0.429
	Bathrooms	0 (0.0)	8 (18.6)	3 (4.2)	5 (4.4)	16	6.0			
	Classrooms	8 (20.5)	28 (65.1)	58 (80.6)	88 (77.9)	182	68.2			
	Corridors	2 (5.1)	4 (9.3)	9 (12.5)	14 (12.4)	29	10.9			
	Playgrounds	24 (61.5)	0 (0.0)	2 (2.8)	5 (4.4)	31	11.6			
	Psychomotricity room	5 (12.8)	3 (7.0)	0 (0.0)	1 (0.9)	9	3.4			
	Total	39 (100)	43 (100)	72 (100)	113 (100)	267	100			

### 3.2. Pattern analysis by Chi-square automatic interaction detection

**Figure 2:**
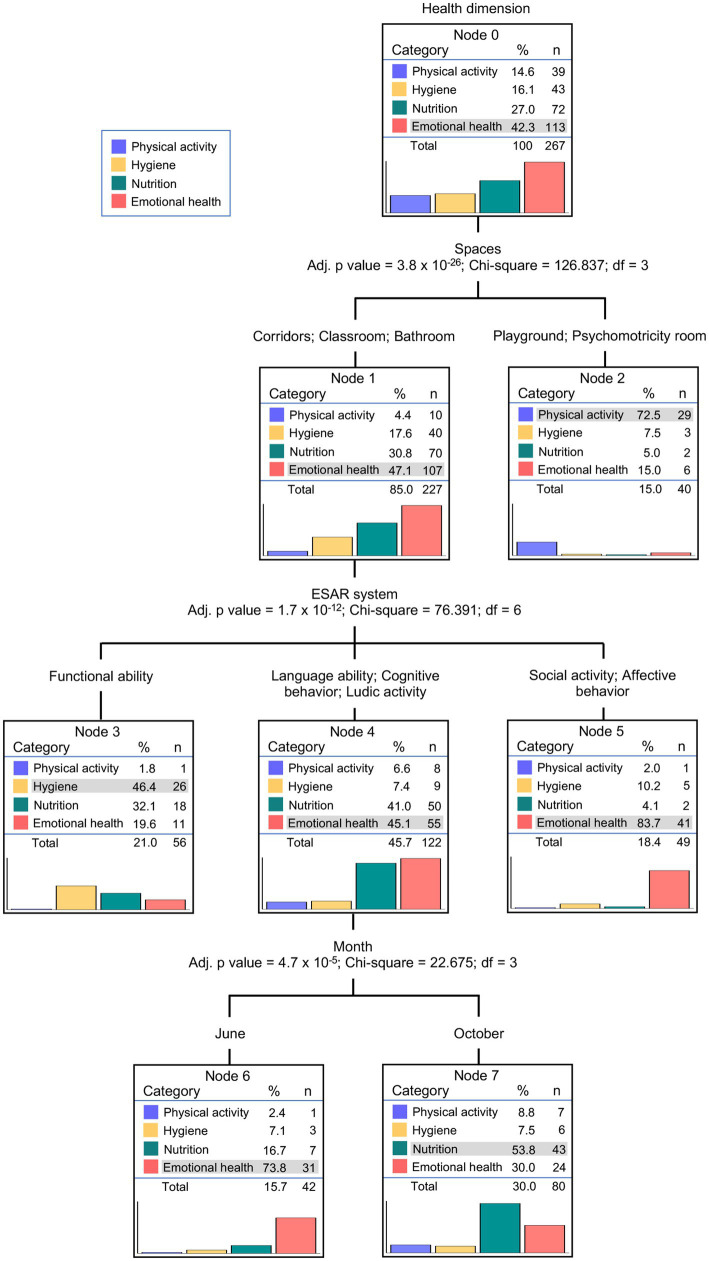
Classification tree using the exhaustive Chi-square automatic interaction detection (CHAID) algorithm to predict the patterns of the pedagogical actions of teachers in the promotion of children’s healthy habits.

To obtain a model for explaining the patterns of pedagogical actions addressed to foster the children’s healthy habits, we performed a classification tree analysis using Health Dimension as the dependent variable and the variables Educational Resource, ESAR Facets, Spaces, Month, and School as predictors. The model generated by CHAID analysis presented a classification tree with 8 nodes, 5 terminal nodes (nodes 2, 3, 5, 6, and 7), and a depth level of 3 (Figure 2).

The factor that best predicted the pedagogical actions and school routines on the four health dimensions was Spaces, which split Node 0 into 2 lines. As defined in terminal Node 2, 72.5% of the pedagogical actions carried out in the playgrounds and psychomotricity rooms were addressed to work Physical Activity, while the other health dimensions were mainly addressed in the classrooms, corridors, and bathrooms (Node 1). The learning facets classified according to the ESAR system presented the second level of prediction and segmented data from Node 1 into 3 new lines. At this level, pedagogical actions addressed to develop Functional Ability were mainly displayed to work the Hygiene dimension (46.4%; terminal Node 3), while the facets of Social Activity and Affective Behavior, were mainly addressed to promote Emotional Health (83.7%; terminal Node 5). At this level, the ESAR learning facets Language Ability, Cognitive Behavior, and Ludic Activity were mainly addressed to work Emotional Health and Nutrition dimensions (Node 4). Finally, the Month of data collection presented the third level of prediction and segmented data from Node 4 into 2 terminal nodes. In June, the pedagogical actions of teachers mainly addressed the Emotional Health dimension (73.8%; terminal Node 7), whereas these actions mainly addressed the Nutrition dimension in October (53.8%, terminal Node 8). The predictors School and Educational Resource did not significantly contribute to the model (adj. *p*-value >0.05 or a number of cases in the node lower than 40) and did not appear in the classification tree.

The generated CHAID model presented a Risk of an estimated 0.363 ± 0.029 based on the estimations of the classification table (Table 2), indicating that 63.7% of the pedagogical actions were correctly classified in the tree. Nutrition is the health dimension with the lowest positive predictive value (a sensitivity of 59.7%) followed by the Hygiene dimension with a predictive value of 60.5% and the Emotional Health dimension with a predictive value of 63.7%. This is consistent with some of the proportions in the terminal nodes of the classification tree. Thus, according to terminal Node 3, pedagogical actions addressed to develop Functional Ability in classrooms, corridors, and bathrooms were mainly displayed to work the Hygiene dimension (46.4%), but it should be noted that 32.1% of these actions were instead addressed to work the Nutrition dimension. Similarly, terminal Node 8 shows that, in October, learning facets of Language Ability, Cognitive Behavior, and Ludic Activity approached in classrooms, corridors, and bathrooms were mainly addressed to the Nutrition dimension (53.8%), while 30.0% of those pedagogical actions were focused on the Emotional Health dimension.

**Table 2:** CHAID classification table.

	Predicted				
Observed	Physical activity	Hygiene	Nutrition	Emotional health	Percent correct
Physical activity	29	1	7	2	74.4%
Hygiene	3	16	6	8	60.5%
Nutrition	2	18	43	9	59.7%
Emotional health	6	11	24	72	63.7%
Overall percentage	15.0%	21.0%	30.0%	34.1%	63.7%

## 4. Discussion

In this article, we define for the first time the pattern of pedagogical actions and interaction trends of preschool teachers to foster healthy habits within children. To characterize this interaction pattern, we first used photography as a strategy for data collection and then applied a data mining analysis by using a CHAID classification tree. With this innovative mixed-methods research approach, we found that the school space and the learning playfulness strategies for the development of executive functions, classified according to the ESAR model, were the two main factors influencing the pedagogical actions of preschool teachers fostering healthy habits.

As a strategy of qualitative data collection, images can offer valuable data on the school activities and environment. In our hands, photography provided evidence about how teachers are working, about their activity patterns and interactions with children, and even captured the school environment providing information on whether it is healthy or not. Photographs emerge as a useful tool to observe the school’s reality from the educational action point of view (Britsch, 2019). Furthermore, they also have a leading role in documenting the children’s voices (Einarsdottir, 2005). Many voices, including teachers, principals, children, and families, can give us the reflection of the health factors generating the environment in the school. But a walk in the classroom can also provide information about the degree of tranquility in the classroom as well as the teacher’s skills. Our study emphasizes determining this environment using photographs. It also provides new evidence on photography capturing data about healthy habits being in-real-time implemented, both in terms of all educational spaces in the whole school (Lewallen et al., 2015), and in terms of the emerging concept of health education integrating physical, mental, and social health with the frailty concept (Boers and Cruz Jentoft, 2015; Gavidia, 2016).

In our methodological approach, we have transformed qualitative information from photographs into quantitative data about the teachers’ pedagogical actions, suitable to be processed statistically in a CHAID analysis. This crossover mixed-method analysis allowed us to derive conclusions and meta-inferences unlikely to reach without this type of analysis (Hitchcock and Onwuegbuzie, 2020). To our knowledge, this is the first study implementing this approach to define the interaction trends of preschool teachers addressed to promote healthy habits within children. Under our conditions, we found a significant pattern of teacher preferences when performing their pedagogical actions. The robustness of our classification model relies on both the criteria of the exhaustive CHAID analysis performed (Önder and Uyar, 2017; Feu et al., 2019; Rodríguez-Sabiote et al., 2021) and on the very low Bonferroni-adjusted *p*-values obtained in the analysis, which are a measure of the compatibility of the entire model of data analysis with the structure of the collected data (Greenland et al., 2016).

School is an excellent setting to promote physical, emotional, and social well-being among students, which also has academic benefits (Pulimeno et al., 2020). Because of that, we have analyzed different educational spaces, such as classrooms, corridors, playgrounds, and bathrooms, following new approaches to health education such as the whole school, whole community, and whole child model (Lewallen et al., 2015). We are struck by how clearly our results point to the weight of spaces of the school in shaping how healthy habits are stimulated, such as those regarding the Physical Activity dimension, which is mostly addressed in playgrounds and psychomotricity rooms. This result illustrates the educational tendency stating that the educator’s role during the children’s free play is mainly as a “facilitator” and then “observer” but not really implicated in the activity (Kalpogianni, 2019). According to new tendencies in education at the playgrounds, such as active and inclusive playgrounds (Angulo et al., 2022), it would be interesting to promote a more involved preschool teacher in the children’s free play. This implies not leaving physical activity only in the playground or in the psychomotricity classroom, but integrating it into the ordinary classroom, since this age represents a critical period to intervene (Baidal and Taveras, 2012; Goldfield et al., 2012). Furthermore, since our study points out that it is beneficial to teach “how to do it,” especially at this young age, it would be enriching if teachers become significant role models in healthy habits alongside families (Natale et al., 2014).

These results also highlight the need to continue working on the widely accepted importance of physical activity in relation to health. Specifically, being physical activity an important factor in the obesogenic environment and a determinant of childhood obesity, highlighting its role in promoting health in recreational or physical activity facilities (Mei et al., 2021). Ideally, physical activity needs to be highly promoted within classrooms and other school spaces. Because it not only contributes to the development of gross motor skills, but also coordination through dance and play, learning to get the body moving, and acquiring healthy routines and habits (Santos-Beneit et al., 2022).

Our results also revealed what learning strategies for the development of executive functions of children are addressed by teachers to promote healthy habits in their educational practice. Furthermore, that pedagogical strategy is the second-best predictor of teacher interventions in the resulting CHAID classification tree. Previous studies report improvement in the ability of executive functions in preschoolers after based intervention (Keown et al., 2020). Thus, the development of executive functions through the promotion and practice in the classroom from early ages both at school and at home is essential. Understanding the way that teachers address these functions is a first step to appropriately defining the educational approaches that best increase children’s healthy behaviors. The ESAR model systematically categorizes these executive actions, which facilitates the comprehension of children’s development (Garon, 1992). Our results revealed this model as a valid categorization of executive actions when classifying the actions carried out by teachers for health promotion in their educational practice. Specifically, the Hygiene dimension of health was mainly promoted in the classroom by addressing the Functional Ability of children, while Social Activity and Affective Behavior were the functions mobilized to address the Emotional Health dimension. Finally, Language Ability, Cognitive Behavior, and Ludic Activity were addressed by teachers mainly to promote Nutrition and also Emotional Health dimensions.

Related to these different pedagogical approaches we also observed a focus of teachers’ actions on addressing Emotional Health, which has a distinct position compared to the other analyzed health dimensions—i.e., Nutrition, Physical Activity, and Hygiene. These results are in line with previous findings showing that the educative community tends to identify health with appropriate Nutrition and considers Emotional health as the most important dimension of health to be developed among preschoolers (Violant-Holz et al., 2021). Taken altogether, our results reflect that teachers address the different health dimensions in separate pedagogical actions in specific school spaces. This points out the pedagogical necessity to develop multidimensional health educational programs (Santos-Beneit et al., 2022), because there is still room for implementing healthy lifestyle habits, and in doing it understanding health promotion as a complex phenomenon (Morin et al., 2002). Aligned with this, we would also like to stress how important is the teacher’s education to develop appropriate teaching skills and strategies (Ferreira et al., 2021), and to see themselves as role models and promoters of healthy habits (Ho and Funk, 2018). Furthermore, especially regarding teachers’ education, we can also see how there is still room for creativity and innovation regarding the didactics of healthy habits at the school (Gómez-García et al., 2020).

On the contrary, we want to draw attention to the fact that the variables Schools and Pedagogical resources do not appear in our CHAID classification tree. Thus, compared to the other factors analyzed, the school and the pedagogical resources of the classroom are factors with a smaller impact on working with healthy habits. This result may be related to a possible homogeneity of the response in the classroom regardless of the teacher, the school, and even each student. Considering that the trend is to move toward an inclusive school model with personalized learning (Generalitat de Catalunya, Departament d’Educació, 2015), this result should encourage the educational community to reflect and debate about health pedagogy in the schools of today and tomorrow.

In addition to its efficiency and accuracy in evaluating potential predictor variables (Collins, 2021), the CHAID analysis also provides a Risk estimate that specifies the proportion of cases classified incorrectly (Mardikyan and Badur, 2011). In our study, the relatively elevated Risk estimate may indicate that these patterns of pedagogical actions are flexible. Research indicates that preschool teachers display differentiated intervention levels, adapt their actions depending on the circumstances of the moment, and provide autonomy support reinforcing children’s self-determination (Sagastui et al., 2020). Under this point of view, the Risk estimate may quantify the multiplicity of pedagogical actions that teachers conduct to address health education through different strategies, in the different school spaces at any time. This is consistent with the whole school, whole community, and whole child model of approaching educational health (Lewallen et al., 2015). Indeed, in our model, teachers’ pedagogical actions addressed the development of Functional Ability in classrooms were mainly displayed to work the Hygiene dimension, but also in no insignificant proportion to work the Nutrition dimension. More interestingly, we observed teachers’ interventions on the Emotional Health dimension mostly in classrooms, corridors, and bathrooms by implementing the Social Activity and Affective Behavior facets of the ESAR model. However, we also documented a non-negligible percentage of actions addressed to this health dimension in other school spaces and ESAR facet interventions. This is in accordance with the high importance that preschool teachers give to the promotion of emotional health among preschoolers (Violant-Holz et al., 2021).

To sum up, in our study, the combination of photography as a resource to collect data with the CHAID classification tree as a quantitative statistical analysis unmasks the inter-dependence relationships between the different factors that determine the teacher’s actions at the school. In our analysis, the school spaces and the learning playfulness strategies for the development of executive functions are the two main factors influencing the pedagogical actions of preschool teachers in fostering healthy habits. Thus, Physical Activity was mainly addressed in the playgrounds and psychomotricity rooms, while it was hardly addressed in regular classrooms. By contrast, we found that the school and the pedagogical resources of the classroom are factors with a much smaller impact on working with healthy habits. These results also teach us that researchers need to be creative in approaching and analyzing what happens in the classroom. Indeed, innovation appears crucial whether we want to improve pedagogical practice based on healthy learning.

### 4.1. Some limitations and possible future lines of research

This study presents some limitations. First, during data collection, the fact that teachers were warned of the presence of observers in advance may influence their practice. However, this was done following the ethics standards in naturalistic observation. Second, data collection in this study is based on a single qualitative method. Thus, data transformation into quantitative variables to be analyzed statistically may be biased due to the interpretive subjectivity of researchers. To minimize the impact of this bias, the classification criteria were first defined, and then, data transformation was conducted by consensus agreement, until mutual exclusion was reached (Arana et al., 2016). Finally, even though this is small and local research, it provides significant results. It would be interesting to replicate this research on a larger scale. In this sense, more locations and more centers would be needed to draw more general classification patterns. It would also be interesting to perform more observations and longer registers.

From the methodological point of view, photography brings us the possibility of analyzing the influence of healthy and unhealthy environments on psychological health (Taylor et al., 1997). This can be true not only for the obesogenic environment (Mei et al., 2021) but also, as shown in our study, for more health dimensions, with a holistic analysis of the environment (Sagala and Munawar, 2018). All in all, photography allows us to see what is happening at a particular time point (Cleland and MacLeod, 2021) and has proven to be an effective method to capture the reality of the schools in education research, for example by the children’s perspectives (Kyrönlampi et al., 2021). In our hands, photography, as a visual method to reflect teacher strategies in the daily life of preschool, and CHAID analysis, as a tool to classify data and unmask relationships, have been proven valuable methods to define a pattern of teachers’ strategies in the daily life of preschool. It will be interesting in the future to combine the use of photography with other observation instruments and data analysis procedures that would complement and consolidate our results.

### 4.2. Conclusion

The present study reaches a series of conclusions. First, we provide new evidence on the value of photography as a visual method of doing social research. Especially regarding its capacity to show an effective way of capturing the school environment and the promotion of healthy habits in the school’s daily life, at the very moment they are being promoted by preschool teachers. Secondly, the ESAR model systematically categorizes the learning playfulness strategies for executive function development, which facilitates the comprehension of children’s development. Thirdly, the school space and the learning playfulness strategies for the development of executive functions, classified according to the ESAR model, are the main factors that influence the pedagogical actions fostering healthy habits. This pedagogical action pattern is flexible, since teachers conduct a multiplicity of pedagogical actions through different strategies, in different school spaces, at any time. Finally, promoting healthy habits and routines in schools requires the integration of multiple variables, such as spaces for pedagogical action, the learning strategies promoted by teachers in these spaces, and the moment at which these habits are being promoted. Applying action pattern analyses will help to better define the factors that influence the promotion of such habits among preschoolers.

## Data availability statement

The datasets generated and analyzed during the current study are not publicly available due to the need for informed consent for participation and bioethics agreement, but they are available from the corresponding author upon reasonable request.

## Ethics statement

Ethical approval of the methods and experimental protocols of this study was granted by the Universitat de Barcelona (Spain). Institutional Review Board approval number-IRB00003099. The study followed the regulations established by the European Union (EU) 2016/679 of the European Parliament and the Council from April 27th on the protection of natural persons with regard to the processing of personal data and free movement of such data, and the Spanish Ley Orgánica 3/2018, from December 5th on the protection of personal data and digital rights. Teachers participated voluntarily in the study and were willing to take photographs and be observed in the classroom in different sessions, at the observer’s choice. All participants and/or legal representatives gave their written informed consent to participate in the study in paper format.

## Author contributions

VV-H conceived the study. All authors collected the data. VV-H and CR-S anonymized data and performed the qualitative analysis. MR performed the analysis of quantitative data. All authors contributed to the article and approved the submitted version.

## Funding

This study is part of all@once and schools@once European research projects (Project numbers: 600137 and 210582, respectively) that received funding from EIT Health. EIT Health is supported by the European Institute of Innovation and Technology (EIT), a body of the European Union that receives support from the European Union’s Horizon 2020 Research and Innovation Programme.

## Conflict of interest

The authors declare that the research was conducted in the absence of any commercial or financial relationships that could be construed as a potential conflict of interest.

The reviewer MA declared a shared affiliation with the authors to the handling editor at the time of review.

## Publisher’s note

All claims expressed in this article are solely those of the authors and do not necessarily represent those of their affiliated organizations, or those of the publisher, the editors and the reviewers. Any product that may be evaluated in this article, or claim that may be made by its manufacturer, is not guaranteed or endorsed by the publisher.
