# Supplementary material for: Preschool teachers display a flexible pattern of pedagogical actions in promoting healthy habits in children
Source: Front Psychol. 2023 Apr 24;14:1172460. doi: 10.3389/fpsyg.2023.1172460 (PMC10165009; doi:10.3389/fpsyg.2023.1172460)
Supplement: Supplementary file 1 [file Table_1.DOCX]

Supplementary Material

Preschool teachers display a flexible pattern of pedagogical actions in promoting healthy habits in children

**Verónica Violant-Holz*, Carlota Rodríguez-Silva, Manuel J Rodríguez***

**Correspondence:**

Corresponding Authors: Verónica Violant-Holz, [vviolant@ub.edu](mailto:vviolant@ub.edu),

Manuel J Rodríguez [marodriguez@ub.edu](mailto:marodriguez@ub.edu)

# Supplementary Table. Deductive checklist establishing the data collection criteria.

| At the school (corridors, playgrounds, bathrooms, psychomotricity rooms) | | | | |
| --- | --- | --- | --- | --- |
|  | Nutrition | | | Y/N |
|  |  | Visible information | | Y/N |
|  |  | Water sources | | Y/N |
|  |  | Healthy food brought from home | | Y/N |
|  | Physical activity | | | Y/N |
|  |  | Visible information | | Y/N |
|  |  | Dangerous spots | | Y/N |
|  | Emotional health | | | Y/N |
|  |  | Visible information | | Y/N |
|  | Hygiene | | | Y/N |
|  |  | Visible information | | Y/N |
| At the regular classroom | | | |  |
|  | Nutrition | | | Y/N |
|  |  | Visible information | | Y/N |
|  | Physical activity | | | Y/N |
|  |  | Visible information | | Y/N |
|  |  | Dangerous spots | | Y/N |
|  | Emotional health | | | Y/N |
|  |  | Visible information | | Y/N |
|  |  | Strategies | | Y/N |
|  |  |  | Resources | Y/N |
|  |  |  | Ludic strategies | Y/N |
|  | Hygiene | | | Y/N |
|  |  | Visible information | | Y/N |
| Entering and leaving the school | | | |  |
|  | Nutrition | | | Y/N |
|  | Physical activity | | | Y/N |
|  |  | Dangerous spots | | Y/N |
|  |  | Daily routines | | Y/N |
|  |  |  | Transport | Y/N |
|  |  |  | Complementary activities | Y/N |
